# Supplementary material for: International External Validation of Risk Prediction Model of 90-Day Mortality after Gastrectomy for Cancer Using Machine Learning
Source: Cancers (Basel). 2024 Jul 5;16(13):2463. doi: 10.3390/cancers16132463 (PMC11240515; doi:10.3390/cancers16132463)
Supplement: Supplementary file 1 [file cancers-16-02463-s001.zip › Supplementary table S1.pdf]

Supplementary Table S1

Country, hospital and city		Number of cases
**ITALY**		**1353**
University of Verona	Verona	407
IEO	Milano	262
San Raffaele Hospital	Milano	240
Morgagni-Pierantoni Hospital	Forlì	207
University of Torino	Torino	90
University of Brescia	Brescia	80
Cattolica University	Roma	67
**POLAND**		**348**
Medical University of Lublin	Lublin	140
Jagiellonian University	Krakow	110
Wroclaw Medical University	Wroclaw	98
**THE NETHERLANDS**		**319**
Academic Medical Center	Amsterdam	175
Netherlands Cancer Institute	Amsterdam	81
Erasmus Medical Center	Rotterdam	63
**PORTUGAL**		**166**
Portuguese Institute of Oncology	Porto	115
University of Lisbon	Lisbon	51
**GERMANY**		**138**
University Hospital of Leipzig	Leipzig	70
Technical University Munich	Munich	60
Agaplesion Markus Hospital	Frankfurt	8
**GREAT BRITAIN**		**110**
St. Thomas NHS	London	69
Royal Marsden NHS	London	41
**SPAIN** [&]		**49**
Hospital del Mar	Barcelona	49
**FRANCE**		**40**
Centre Hospitalier Régional	Lille	40
**IRELAND**		**39**
Trinity College Dublin	Dublin	39
**SWITZERLAND**		**35**
University Hospital of Geneva	Geneva	23
Hirslanden Medical Center	Zurich	12

[&] all cases from the Hospital del Mar registered in the GASTRODATA registry were excluded.
