# Supplementary material for: International External Validation of Risk Prediction Model of 90-Day Mortality after Gastrectomy for Cancer Using Machine Learning
Source: Cancers (Basel). 2024 Jul 5;16(13):2463. doi: 10.3390/cancers16132463 (PMC11240515; doi:10.3390/cancers16132463)
Supplement: Supplementary file 1 [file cancers-16-02463-s001.zip › Supplementary table S2.pdf]

Supplementary Table S2. Performance Metrics from the cv-Enet, glmboost and Ensemble Models in the External Validation Cohort

Metrics	cv-Enet	glmboost	ensemble
AUC	0.659 [0.605-0.713]	0.687 [0.636-0.738]	0.701 [0.647-0.755]
Sensitivity	0.200 [0.125-0.295]	0.232 [0.151-0.329]	0.389 [0.291-0.495]
Specificity	0.899 [0.886-0.910]	0.891 [0.878-0.904]	0.862 [0.847-0.875]
PPV	0.071 [0.043-0.109]	0.076 [0.048-0.113]	0.098 [0.070-0.133]
NPV	0.967 [0.958-0.974]	0.968 [0.960-0.975]	0.973 [0.966-0.980]
AUPRC	0.070	0.081	0.086
